# Satellite-Based Estimates of Long-Term Exposure to Fine Particles and Association with Mortality in Elderly Hong Kong Residents

**DOI:** 10.1289/ehp.1408264

**Published:** 2015-04-24

**Authors:** Chit Ming Wong, Hak Kan Lai, Hilda Tsang, Thuan Quoc Thach, G. Neil Thomas, Kin Bong Hubert Lam, King Pan Chan, Lin Yang, Alexis K.H. Lau, Jon G. Ayres, Siu Yin Lee, Wai Man Chan, Anthony J. Hedley, Tai Hing Lam

**Affiliations:** 1 School of Public Health, The University of Hong Kong, Hong Kong, China; 2 School of Health and Population Sciences, University of Birmingham, Edgbaston, Birmingham, United Kingdom; 3 Nuffield Department of Population Health, University of Oxford, Oxford, United Kingdom; 4 School of Nursing, The Hong Kong Polytechnic University, Hong Kong, China; 5 Division of Environment, The Hong Kong University of Science and Technology, Hong Kong, China; 6 Department of Health, the Government of Hong Kong, Hong Kong, China

## Abstract

**Background:**

A limited number of studies on long-term effects of particulate matter with aerodynamic diameter < 2.5 μm (PM_2.5_) on health suggest it can be an important cause of morbidity and mortality. In Asia where air quality is poor and deteriorating, local data on long-term effects of PM_2.5_ to support policy on air quality management are scarce.

**Objectives:**

We assessed long-term effects of PM_2.5_ on the mortality in a single Asian city.

**Methods:**

For 10–13 years, we followed up a cohort of 66,820 participants ≥ 65 years of age who were enrolled and interviewed in all 18 Elderly Health Centres of the Department of Health, Hong Kong, in 1998–2001. Their residential addresses were geocoded into *x*- and *y*-coordinates, and their proxy exposures to PM_2.5_ at their addresses in 1 × 1 km grids were estimated from the U.S. National Aeronautics and Space Administration (NASA) satellite data. We used Cox regression models to calculate hazard ratios (HRs) of mortality associated with PM_2.5_.

**Results:**

Mortality HRs per 10-μg/m^3^ increase in PM_2.5_ were 1.14 (95% CI: 1.07, 1.22) for all natural causes, 1.22 (95% CI: 1.08, 1.39) for cardiovascular causes, 1.42 (95% CI: 1.16, 1.73) for ischemic heart disease, 1.24 (95% CI: 1.00, 1.53) for cerebrovascular disease, and 1.05 (95% CI: 0.90, 1.22) for respiratory causes.

**Conclusions:**

Our methods in using NASA satellite data provide a readily accessible and affordable approach to estimation of a sufficient range of individual PM_2.5_ exposures in a single city. This approach can expand the capacity to conduct environmental accountability studies in areas with few measurements of fine particles.

**Citation:**

Wong CM, Lai HK, Tsang H, Thach TQ, Thomas GN, Lam KB, Chan KP, Yang L, Lau AK, Ayres JG, Lee SY, Chan WM, Hedley AJ, Lam TH. 2015. Satellite-based estimates of long-term exposure to fine particles and association with mortality in elderly Hong Kong residents. Environ Health Perspect 123:1167–1172; http://dx.doi.org/10.1289/ehp.1408264

## Introduction

Among the World Health Organization criteria pollutants, particulate matter (PM) is often considered the most policy relevant because it is emitted from burning of fossil fuels in road traffic, shipping, and power generation, and it affects almost all organ systems of the body (WHO 2005). In particular, evidence from animal studies, but not yet confirmed in humans, shows that particles with aerodynamic diameter < 2.5 μm (PM_2.5_) can enter the blood stream via the alveolar capillaries, with the potential to cause serious health problems (Anderson et al. 2012).

Studies of long-term effects of PM_2.5_ have typically focused on mortality, and are mostly from North America and Europe. Findings have been inconsistent, with American studies reporting associations with cardiovascular diseases (Crouse et al. 2012; Krewski et al. 2009), and European studies reporting associations with respiratory diseases (Beelen et al. 2014; Carey et al. 2013).

To our knowledge, there are seven published studies on long-term effects of air pollution on mortality in Asia (Cao et al. 2011; Dong et al. 2012; Katanoda et al. 2011; Nishiwaki et al. 2013; Ueda et al. 2012; Zhang et al. 2011; Zhou et al. 2014). Among them only one study assessed the effects of PM_2.5_, but due to a lack of direct measurements, PM_2.5_ was estimated from PM_10_ (PM < 10 μm in aerodynamic diameter) by means of a presumed ratio of PM_2.5_/PM_10_ (Katanoda et al. 2011).

Mitigation of air pollution is urgently needed in Asia, where air quality has deteriorated quickly due to rapid industrialization and urbanization (Hedley et al. 2008). In the present study, we estimated PM_2.5_ with a novel method using high-resolution satellite data from the National Aeronautics and Space Administration (NASA) (Paciorek et al. 2012) and assessed long-term effects on mortality in a large elderly cohort in Hong Kong, the most economically developed city in China.

## Methods

We included a total of 66,820 participants ≥ 65 years of age who enrolled in an Elderly Health Centre of the Department of Health in one of the 18 districts of Hong Kong in 1998–2001, and we examined mortality outcomes through record linkage to the death registry until 31 December 2011. Socioeconomic factors, lifestyle characteristics, and morbidity status were collected by face-to-face interview during enrollment and follow-up visits by registered nurses (Lam et al. 2004). All participants provided informed consent. Ethics approval was obtained from the Ethics Committee of the Faculty of Medicine, The University of Hong Kong.

In Hong Kong there are no zip codes or postal codes. We geocoded addresses for all participants onto an area map with demarcation of areas of District Boards and Tertiary Planning Units (TPU) for which ecological level sociodemographic variables could be obtained from the 2001 Census (Census and Statistics Department, Government of the Hong Kong Special Administrative Region 2002).

Aerosol optical depth (AOD) retrieved from remote sensing data of the two NASA Earth Observing System satellites is a measure of transparency for electromagnetic radiation as well as an indication of PM levels in the troposphere (NASA 2013). AOD data are originally retrieved in 10 × 10 km resolution, but with a 99% cloud-free local environment and adjustment for local meteorological conditions, they can be refined into 1 × 1 km resolution, providing a stronger correlation with PM than the original resolution (Li et al. 2005). We used surface extinction coefficients (SEC) for measuring AOD within 1 km of ground level to predict PM_2.5_ (Division of Environment, The Hong Kong University of Science and Technology 2012). We regressed the annual SEC on annual PM_2.5_ of the four Hong Kong Environmental Protection Department monitors, which measured the pollutant in the period 2000–2011 (see Supplemental Material, Figure S1). For each year, annual PM_2.5_ exposures at geographical locations of individual participants were estimated using the same regression equation, with the annual SEC as the explanatory variable.

Missing SEC data (15.7%), which were mainly due to cloud cover problems (usually occurred from February to May), were filled in by the predicted mean matching method in multiple imputation using the MI procedure in SAS 9.2 (SAS Institute Inc., Cary, NC, USA). Missing data for individual-level covariates were recovered if they were reported in later years; otherwise, the participants were excluded case-wise.

We calculated hazard ratios for deaths categorized according to the *International Classification of Diseases, 10th Revision* (ICD-10; WHO 2010): all deaths from natural causes (codes A00–R99); cardiovascular diseases (I00–99) with subcategories of ischemic heart disease (IHD; I20–25) and cerebrovascular disease (I60–69); respiratory diseases (J00–47, 80–99) with subcategories of pneumonia (J12–18) and chronic obstructive pulmonary disease (COPD; J40–44, 47); and external causes (S00–T99). Study participants were excluded from analyses if they died within 1 year of enrollment or died from a cause other than the one being modeled.

We categorized the participants into four quartiles (Q1–Q4) of PM_2.5_ exposure and plotted the survival curve of mortality from all natural causes for each group using the Kaplan Meier method. We adopted Cox proportional hazard models for survival, with the time scale setting as duration from year of recruitment to the year of death for the causes being modeled or censored at the year of the follow-up in 2011. The independent variable was exposure to average PM_2.5_ at the baseline. Model covariates included individual-level demographic, socioeconomic, and lifestyle factors obtained from interviews; TPU-level sociodemographic variables obtained from the 2001 Census; and district-level data including the proportion of smokers (> 15 years of age) from 1998 to 2011 (Census and Statistics Department, Government of the Hong Kong Special Administrative Region 2011). Individual-level variables in the final model were age (continuous), sex, body mass index (BMI; < 21.6, 21.6–26.3, > 26.3 kg/m^2^), smoking (never, ex-smoker, current smoker), physical exercise (days per week), education (< primary, primary, ≥ secondary), and monthly expenses (< 128, 128–384, > 384 US$). In addition we adjusted for the TPU-level proportion of the population ≥ 65 years of age, the proportion with > secondary education and the average monthly income in each TPU. Finally, we adjusted for the proportion of smokers in each district.

We performed sensitivity analyses using yearly exposures to average PM_2.5_, inclusion of participants who died during the first year after enrollment, and exclusion of participants who died in the first 1–3 years. We also performed separate analyses stratified by age (< 71 or ≥ 71 years, based on the median age of 70), sex, and education (< primary, or ≥ primary). In addition, we stratified according to the length of follow-up (2 to < 5, 5 to < 9, or ≥ 9 years after baseline). We also used models with random effects set at the intercepts to take account of possible intradistrict correlations and with an extension to take account of spatial autocorrelations due to adjacency or distance decay between TPUs (Burnett et al. 2001; Ma et al. 2003).

All statistical analyses were performed using Stata 10.0 (StataCorp., College Station, TX, USA) and SAS 9.2.

## Results

**Figure 1:**
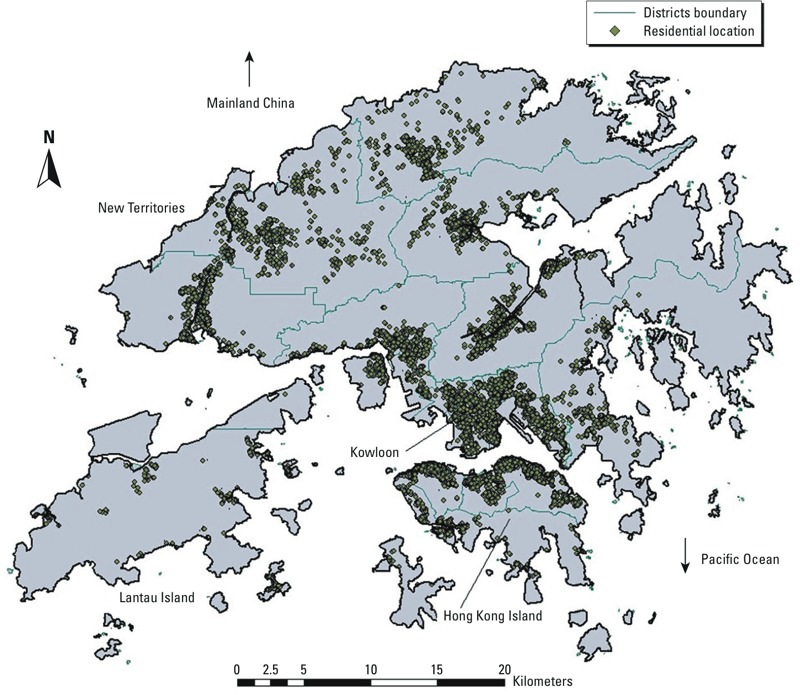
Spatial distribution of geocoded addresses of participants and boundaries of the 18 districts (*n* = 60,221). Each district has one Elderly Health Centre to provide health service for persons ≥ 65 years of age who have enrolled voluntarily. Those enrolled in 1998–2001 were recruited to this study, and their residential addresses were geocoded into *x*- and *y*-coordinates, which fell into 1 × 1 km grids on the Hong Kong map.

A total of 66,820 participants were recruited, with 64,888 addresses geocoded (97%); the participants were distributed over the 18 districts of Hong Kong (Figure 1) and accounted for 6.5–17.2% of the population ≥ 65 years of age. After the exclusion of missing data due to missing individual-level covariates (0.2%), problems in geocoding (8.1%), or problems in satellite data (1.5%), a final sample of 60,221 participants (90.1%) was included for PM_2.5_ estimation and analysis.

**Figure 2:**
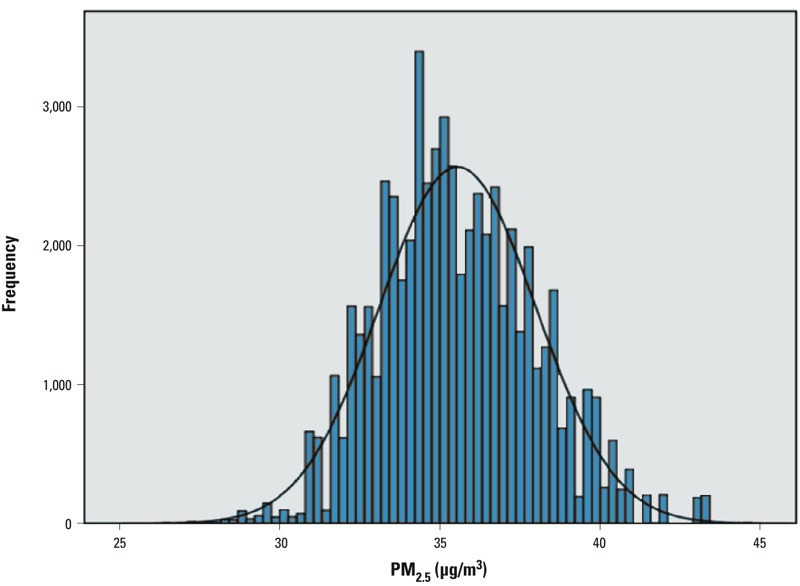
Distribution of PM_2.5_ estimated at geocoded addresses of participants (*n* = 59,591). The width of each bar (*x*-axis) represents a class interval for a range of PM_2.5_ exposure proxy of individuals, and the height (*y*-axis) represents the frequency of addresses in that class interval.

During the study period, PM_2.5_ was in general increasing, but there were ups and downs in some years. Such patterns were similar across geographic areas (data not shown). At the baseline, the estimated concentrations of PM_2.5_ approximated to a normal distribution (Figure 2), approximately 70% of the participants were 65–74 years of age, and 65% were female. Participants in the highest exposure category (Q4) tended to be older and were more likely to be smokers and habitually exercise less, less likely to have secondary or higher education, and more likely to have higher levels of personal expenditure, compared with those with lower exposure (Q1–Q3) (Table 1). TPUs with a higher concentration of PM_2.5_ tended to be associated with lower household income (Spearman correlation: –0.155; *p* = 0.030) (data not shown).

**Table 1:** Descriptive statistics of the participants by four categories by quartiles of PM_2.5_ concentration derived from the NASA satellite data (*n* = 59,591*^a^*).

Variable	PM_2.5_ category^*b*^			
	Q1	Q2	Q3	Q4
PM_2.5_ concentration (μg/m^3^, mean ± SD)	32.6 ± 1.03	34.6 ± 0.43	36.2 ± 0.53	38.8 ± 1.34
Participants (*n*)	14,907	15,167	14,684	14,833
Incidence rate for all deaths (per 100,000 person-years)	447	461	462	495
Individual level
Age (years, mean ± SD)	71.8 ± 5.4	71.9 ± 5.5	71.8 ± 5.5	72.2 ± 5.5
Sex (%)
Male	34.7	33.7	34.9	33.6
Female	65.3	66.4	65.1	66.4
BMI quartile (%)
2nd–3rd (21.6–26.3 kg/m^2^)	50.0	50.6	51.0	51.6
1st (< 21.6 kg/m^2^)	21.6	23.5	22.3	24.0
4th (> 26.3 kg/m^2^)	28.4	25.9	26.7	24.4
Smoking (%)
Never	71.8	71.5	70.9	70.5
Quit	18.7	19.0	19.6	19.5
Current	9.5	9.5	9.5	10.0
Exercise
Days per week (mean ± SD)	5.6 ± 2.5	5.5 ± 2.6	5.4 ± 2.7	5.4 ± 2.7
Education (%)
≥ Secondary	17.6	18.4	17.8	15.3
Primary	34.6	37.4	38.3	38.3
< Primary	47.8	44.2	43.9	46.4
Expense per month (US$, %)
< 128	17.0	15.7	14.0	12.4
128–384	69.7	70.5	67.7	67.7
≥ 385	13.3	13.8	18.3	19.9
TPU level**(mean percent ± SD)
≥ 65 years of age	11.4 ± 4.2	11.5 ± 3.8	12.0 ± 3.9	13.6 ± 4.4
> Secondary education	13.7 ± 8.5	13.4 ± 8.1	13.3 ± 8.1	11.8 ± 7.0
Income ≥ 1,923 US$/month	63.3 ± 11.0	61.1 ± 10.5	58.9 ± 11.3	55.1 ± 12.0
District level
Smoking rate (mean percent of smokers ± SD)	11.5 ± 0.4	11.6 ± 0.4	11.6 ± 0.4	11.6 ± 0.3
TPU, Tertiary Planning Units. ^***a***^Of the total 60,221 participants included in the study, 630 had a PM_2.5_ estimate only at baseline. ^***b***^PM_2.5_ concentrations (μg/m^3^): minimum, 26.4; 25th percentile, 33.8; 50th percentile, 35.3; 75th percentile, 37.2; maximum, 44.6. Thus, Q1: 25th percentile; Q2: 25th–50th percentile; Q3: 50th–75th percentile; Q4: > 75th percentile.				

After 10–13 years of follow-up before excluding the 204 deaths in the first year, there were 16,006 deaths from natural causes and 409 from external causes (Table 2). Survival was highest among residents in the lowest exposure group (Q1), slightly lower for those in Q2 and Q3, but markedly lower for those in the highest quartile of exposure (see Supplemental Material, Figure S2).

**Table 2:** Mortality outcomes after 10–13 years of follow up at end of study in 2011.

ICD-10 codes	Mortality cause	No. of deaths	Percent
A00–R99	All natural causes	16,006	97.5
I00–99	Cardiovascular	4,656	28.4
I20–I25	IHD	1,810	11.0
I60–69	Cerebrovascular	1,621	9.9
J00–47, 80–99	Respiratory	3,150	19.2
J12–18	Pneumonia	2,057	12.5
J40–44, 47	COPD	940	5.7
S00–T99	External causes	409	2.5
All included codes	All causes	16,415	

**Figure 3:**
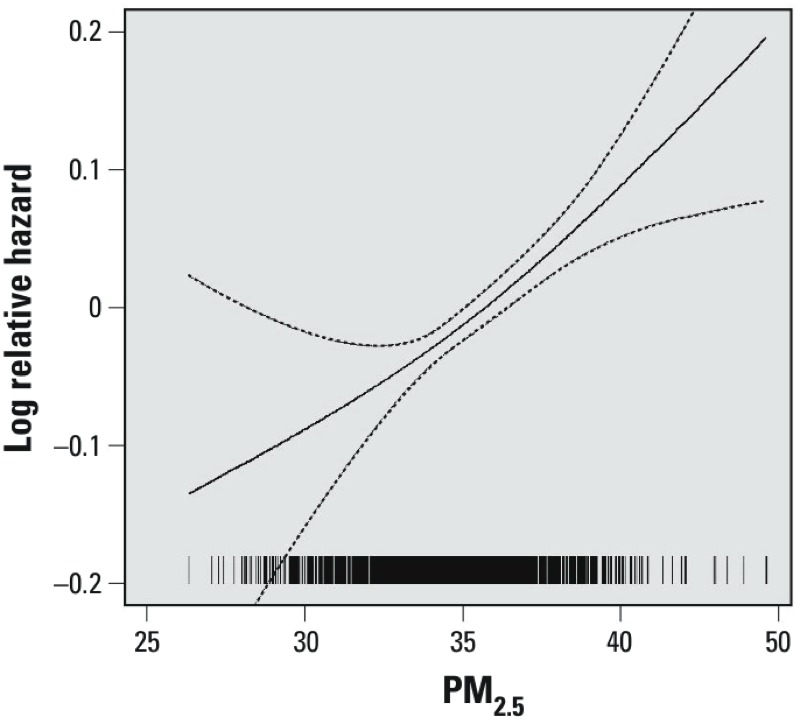
Concentration–response relationship between PM_2.5_ exposure and all natural cause mortality. The figure demonstrates the relative risk (fully adjusted model) of all natural cause mortality in relation to long-term exposure to PM_2.5_. The tick marks on the *x*-axis represent the position of PM_2.5_ concentrations measured in μg/m^3^. Dashed lines represent 95% CI (*p*-value = 0.772 for log likelihood Chi-square test for linear vs. nature spline model).

Before adjusting for any covariates, the HR for all-cause mortality in association with a 10-μg/m^3^ increase in PM_2.5_ was 1.23 [95% confidence interval (CI): 1.16, 1.31]. After adjusting for individual-level covariates only, the HR was 1.13 (95% CI: 1.06, 1.21), whereas the HR from the fully adjusted model (including TPU and district-level covariates) was 1.14 (95% CI: 1.07, 1.22) (see Supplemental Material, Table S1). A natural spline model of the association between PM_2.5_ and all-cause mortality (fully adjusted model) confirmed that the association was linear (*p*-value comparing the fit of the spline model to a linear model = 0.8) (Figure 3).

A 10-μg/m^3^ increase in PM_2.5_ was associated with all cardiovascular disease (HR = 1.22; 95% CI: 1.08, 1.39) and with the subcategories IHD (HR = 1.42; 95% CI: 1.16, 1.73) and cerebrovascular disease (HR = 1.24; 95% CI: 1.00, 1.53) (Table 3). Associations with respiratory mortality and COPD were positive but not statistically significant (HR = 1.05; 95% CI: 0.90, 1.22 and HR = 1.30; 95% CI: 0.98, 1.74, respectively).

**Table 3:** Hazard ratio (95% CI) per 10-μg/m^3^ increase of PM_2.5_ in main analysis for average exposure at the baseline period and sensitivity analyses for exposure to average PM_2.5_ yearly and for different inclusion and exclusion criteria.

Cause of death	Main analysis^*a*^: baseline exposure (*n* = 59,362)	Yearly exposure (*n* = 59,421)	Including deaths within 1 year: baseline exposure (*n* = 59,566)	Excluding deaths within 3 years: baseline exposure (*n* = 57,405)
All natural causes	1.14 (1.07, 1.22)***	1.11 (1.03, 1.20)**	1.14 (1.07, 1.22)***	1.15 (1.08, 1.24)***
Cardiovascular	1.22 (1.08, 1.39)**	1.15 (1.00, 1.33)	1.23 (1.08, 1.39)**	1.19 (1.04, 1.36)*
IHD	1.42 (1.16, 1.73)***	1.40 (1.12, 1.76)**	1.43 (1.17, 1.74)***	1.40 (1.13, 1.73)**
Cerebrovascular	1.24 (1.00, 1.53)*	1.15 (0.91, 1.46)	1.24 (1.01, 1.53)*	1.18 (0.94, 1.47)
Respiratory	1.05 (0.90, 1.22)	1.06 (0.89, 1.26)	1.02 (0.87, 1.18)	1.01 (0.86, 1.19)
Pneumonia	0.94 (0.77, 1.14)	1.02 (0.82, 1.26)	0.92 (0.76, 1.11)	0.91 (0.75, 1.11)
COPD	1.30 (0.98, 1.74)	1.26 (0.92, 1.73)	1.32 (1.00, 1.74)	1.28 (0.95, 1.73)
External causes	1.04 (0.69, 1.58)	1.09 (0.69, 1.75)	1.03 (0.69, 1.56)	1.10 (0.71, 1.72)
^***a***^Deaths within 1 year were excluded. *p < 0.05. **p < 0.01. ***p < 0.001.				

In sensitivity analyses for all natural causes and for overall and subcategories of cardiovascular mortality (Table 3), the estimates and levels of statistical significance remained similar except for cerebrovascular mortality, where the estimates became nonsignificant when yearly average concentration were used as the exposure or when deaths within 1–3 years were excluded. The comparisons between the main and sensitivity analyses for respiratory mortality showed similar levels of estimates and significance.

In stratified analyses (Table 4), the association between a 10-μg/m^3^ increase in PM_2.5_ and mortality was closer to the null (or essentially null) in the ≥ 71 age group compared with the < 71 age group for all mortality outcomes, with significant differences by age for cardiovascular mortality (HR = 1.15; 95% CI: 1.00, 1.33 vs. HR = 1.42; 95% CI: 1.10, 1.84; interaction *p*-value 0.04) and IHD (HR = 1.22; 95% CI: 0.96, 1.53 vs. HR = 2.20; 95% CI: 1.47, 3.29; interaction *p*-value 0.002). Differences were also pronounced for COPD (HR = 1.13; 95% CI: 0.81, 1.57 vs. HR = 2.20; 95% CI: 1.26, 3.86; interaction *p*-value 0.06). There was little evidence of consistent differences in associations between PM_2.5_ and any of the outcomes according to sex (Table 4) or education (see Supplemental Material, Table S2).

**Table 4:** Hazard ratio (95% CI) per 10-μg/m^3^ increase of PM_2.5_ in stratified analyses by age or sex with exposure at baseline (deaths within the first year were excluded).

Cause of death	Age < 71 years	Age ≥ 71 years	Interaction^*a*^	Male	Female	Interaction^*a*^
All natural causes	1.23 (1.08, 1.40)**	1.11 (1.03, 1.19)**	0.07	1.17 (1.06, 1.29)**	1.13 (1.03, 1.23)**	0.7
Cardiovascular	1.42 (1.10, 1.84)**	1.15 (1.00, 1.33)	0.04	1.28 (1.05, 1.57)*	1.19 (1.01, 1.40)*	0.5
IHD	2.20 (1.47, 3.29)***	1.22 (0.96, 1.53)	0.002	1.39 (1.02, 1.90)*	1.44 (1.10, 1.89)**	0.8
Cerebrovascular	1.21 (0.80, 1.84)	1.24 (0.97, 1.59)	0.8	1.32 (0.93, 1.87)	1.21 (0.92, 1.58)	0.9
Respiratory	1.37 (0.96, 1.95)	0.98 (0.83, 1.16)	0.1	1.11 (0.90, 1.38)	0.97 (0.78, 1.22)	0.5
Pneumonia	1.03 (0.63, 1.69)	0.91 (0.74, 1.13)	0.6	1.00 (0.75, 1.32)	0.88 (0.68, 1.15)	0.7
COPD	2.20 (1.26, 3.86)	1.13 (0.81, 1.57)	0.06	1.21 (0.85, 1.72)	1.53 (0.93, 2.52)	0.6
External causes	0.84 (0.41, 1.73)	1.14 (0.68, 1.90)	0.8	1.03 (0.60, 1.77)	1.07 (0.57, 1.99)	0.9
^*a*^*p*-Value for the interaction term in the model for the combined data set. *p < 0.05. **p < 0.01. ***p < 0.001.						

Stratifying for different periods of follow-up (Table 5), the HR for a 10-μg/m^3^ increase in PM_2.5_ and all natural cause mortality was highest for deaths 2–4 years after baseline (HR = 1.32; 95% CI: 1.11, 1.56), and lower for deaths within 5–8 years (HR = 1.12; 95% CI: 1.00, 1.25) and after ≥ 9 years (HR = 1.09; 95% CI: 0.99, 1.19). Mortality for cardiovascular disease and the subcategories IHD and cerebrovascular disease followed a similar pattern. In mortality for respiratory disease and the subcategories pneumonia and COPD, the HRs were markedly high in the first period and were much lower in the second and third periods.

**Table 5:** Hazard ratio (95% CI) per 10-μg/m^3^ increase of PM_2.5_ in stratified analyses by period of follow-up.

Cause of death	2–4 years	5–8 years	≥ 9 years
All natural causes	1.32 (1.11, 1.56)**	1.12 (1.00, 1.25)	1.09 (0.99, 1.19)
Cardiovascular	1.81 (1.32, 2.50)***	1.16 (0.92, 1.45)	1.11 (0.93, 1.32)
IHD	2.36 (1.42, 3.93)**	1.06 (0.73, 1.54)	1.43 (1.09, 1.89)*
Cerebrovascular	1.64 (0.94, 2.87)	1.39 (0.96, 2.00)	1.07 (0.79, 1.43)
Respiratory	1.72 (1.09, 2.73)*	1.07 (0.81, 1.41)	0.93 (0.76, 1.14)
Pneumonia	1.42 (0.72, 2.79)	1.03 (0.72, 1.47)	0.86 (0.67, 1.10)
COPD	2.30 (1.15, 4.63)*	1.16 (0.71, 1.91)	1.12 (0.76, 1.67)
External cause	0.97 (0.44, 2.17)	0.80 (0.38, 1.66)	1.30 (0.69, 2.46)
*p < 0.05. **p < 0.01. ***p < 0.001.			

Estimates from multilevel models that account for clustering at the TPU and district levels were similar to the main analysis (see Supplemental Material, Table S3), as were estimates from models that account for spatial autocorrelation (see Supplemental Material, Table S4).

## Discussion

In Hong Kong, a highly dense subtropical city (2001 population of 6.7 million, land area of 1,104 km^2^), exposure to PM_2.5_ was significantly associated with mortality from natural and cardiovascular causes, and with mortality due to IHD and cerebrovascular disease specifically, in people ≥ 65 years of age. The findings with adjustment for potential confounding factors measured at individual and ecological levels are, in general, robust to different periods of exposure measurement and inclusion and exclusion criteria. The results of our study from a satellite-based measure of PM_2.5_ provide new evidence on mortality from long-term effects of PM_2.5_.

We used a novel strategy to estimate exposure of individuals to PM_2.5_ concentrations based on SEC estimated from AOD data within 1 km of ground level captured by NASA satellites. With improved resolution of 1 × 1 km, SEC is correlated (*r* = 0.6) with the PM_2.5_ concentration measured at the monitors (see Supplemental Material, Figure S1). In one of the few studies using NASA satellite data in Canada, with 10 × 10 km resolution, similar correlations were obtained, and HRs for 10-μg/m^3^ increase in PM_2.5_ were 1.15 (95% CI: 1.13, 1.16) for all natural cause mortality and 1.31 (95% CI: 1.27, 1.35) for IHD (Crouse et al. 2012), consistent with our findings for the south China city of Hong Kong. These results demonstrate the feasibility of using satellite data to derive a valid proxy measure for individual long-term exposure to PM_2.5_ in a typical Asian city with high population density in areas with complicated terrain. The resolution of 1 × 1 km would be adequate to define the exposure of older persons who are most likely retired and confined to within 0.5 km of their place of residence (Chau et al. 2002). In Hong Kong, pollution levels are generally heterogeneous from a public health risk perspective; therefore, while our approach is applicable to an older population with limited mobility, it may be less so for very mobile populations.

The HR estimates of mortality from all natural, cardiovascular, and respiratory causes are consistent with those reported in a recent review (Hoek et al. 2013) and with the combined analysis of 22 European cohorts within the ESCAPE project (Beelen et al. 2014). In an extended analysis of the American Cancer Society study (Krewski et al. 2009), in the nationwide assessment the HR of mortality from all causes was lower than ours; in Los Angeles, California, the HR was similar to ours, but in New York City (NewYork) the HR of around unity was lower than ours. However, for IHD, the estimates were lower than ours. In two other studies published after the review (Hoek et al. 2013), the estimates for respiratory mortality were different and higher (Beelen et al. 2014; Carey et al. 2013; Katanoda et al. 2011). The heterogeneity in effect sizes for respiratory mortality among studies may be due to the different local polluting sources, particularly from traffic. Because the age range (25–85 years) in these studies is broad, the heterogeneity may also be due to the differences in susceptibility. In a previous study we observed that the relative reduction in respiratory mortality was greater in the 15- to 64-year age group than in the ≥ 65-year group after restrictions on sulfur content of fuel in Hong Kong, suggesting heterogeneity of air pollution effects among age groups (Hedley et al. 2002).

In our stratified analysis, participants recruited at ≥ 71 years of age were potentially at lower risk from air pollution than those recruited at < 71 years, probably due to a healthy survivor effect. Particularly for older people, caution is needed in interpreting mortality effects in long study periods, which may vary because of changes in susceptibility of the survivors in different periods of follow-up. Our results show no sex differences. Indeed, the current evidence for sex differences in susceptibility is weak and inconsistent among studies. For example, the American Cancer Society study (Krewski et al. 2009) and the Netherlands study (Beelen et al. 2008) showed a higher risk in females, but the Harvard Six City study showed a lower risk in females for cardiovascular mortality associated with PM_2.5_ (Pope et al. 2002).

Long-term PM_2.5_ exposure was positively associated with all respiratory and COPD mortality, particularly with strong associations for the latter, during the first 2–4 years of follow-up (data not shown). In contrast, PM_2.5_ exposure was not associated with pneumonia mortality. Although long-term exposure to air pollutants is likely to be a key determinant of a person’s susceptibility to viral and bacterial infections, other factors such as health service accessibility could be equally important (Neupane et al. 2010).

After the first coordinated project among four Asian cities in early 2000 (Wong et al. 2008), several studies of the short-term effects of air pollution in Asia have been reported (Balakrishnan et al. 2013; Bae and Park 2009; Chen et al. 2012; Mahiyuddin et al 2013; Rajarathnam et al. 2011; Wong et al. 2008) showing that the effect estimates for PM_10_ are, in general, comparable to those from North America and Europe (Samoli et al. 2008). Short-term effects are limited to health outcomes, which are responsive to short periods of exposure (Künzli et al. 2001).

In recent years, China has been undergoing a stage of transition from mainly economic development to include issues in the environment, for which tighter air quality standards are needed (Chen et al. 2011). With air movements over China, air pollution from the highly polluted northern cities could affect the southern cities, and joint efforts among cities are needed to combat the problems of air pollution. Reliable estimates of health effects of air pollution from epidemiologic studies are urgently needed to provide important scientific evidence for environmental accountability as well as for health impact assessment of new air quality objectives. Our study can fill an important gap in missing long-term effect estimates for Asia and the impact on life expectancy and value of life years, which could be gained due to reduction in the pollutant as a result of government intervention (Hedley et al. 2002). These estimates can form the basis of essential public health information, including communication of the risks of air pollution and supporting the benefit–cost ratios of achieving clean air.

There are some limitations of our study. First, the participants were self-selected for enrollment in the care centers; thus our study was likely to have included health-conscious participants who were less susceptible than those of the general population. Second, because the participants were ≥ 65 years of age on recruitment, the study could not assess health problems that affect younger people. Third, occupational exposures and those experienced before the baseline were not measured, which might have led to bias in estimation of the health effects. Last but not least, the data we used for verifying the estimation model for PM_2.5_ were directly measured by four Hong Kong Environmental Protection Department monitors. A better assessment could be carried out by setting up and measuring the whole area of Hong Kong using a sufficient number of monitors.

## Conclusion

In an observation window of 10–13 years for a population-based cohort of ≥ 65 years of age, exposure to PM_2.5_ estimated from NASA satellite data at the area of residence was associated with mortality for all natural and cardiovascular causes. The effect estimates corroborate the existing evidence for a causal relationship between PM_2.5_ and adverse health outcomes, and support formulation and implementation of policies for the mitigation of the pollutant and its disease burden.

## Supplemental Material

(1.2 MB) PDF

## References

[r1] Anderson JO, Thundiyil JG, Stolbach A (2012). Clearing the air: a review of the effects of particulate matter air pollution on human health.. J Med Toxicol.

[r2] Bae HJ, Park J (2009). Health benefits of improving air quality in the rapidly aging Korean society.. Sci Total Environ.

[r3] Balakrishnan K, Ganguli B, Ghosh S, Sambandam S, Roy SS, Chatterjee A (2013). A spatially disaggregated time-series analysis of the short-term effects of particulate matter exposure on mortality in Chennai, India.. Air Qual Atmos Health.

[r4] Beelen R Hoek G van den Brandt PA Goldbohm RA Fischer P Schouten LJ 2008 Long-term effects of traffic-related air pollution on mortality in a Dutch cohort (NLCS-AIR study). Environ Health Perspect 116 196 202; 10.1289/ehp.10767 18288318 PMC2235230

[r5] Beelen R, Raaschou-Nielsen O, Stafoggia M, Andersen ZJ, Weinmayr G, Hoffmann B (2014). Effects of long-term exposure to air pollution on natural-cause mortality: an analysis of 22 European cohorts within the multicentre ESCAPE project.. Lancet.

[r6] Burnett R, Ma R, Jerrett M, Goldberg MS, Cakmak S, Pope CA (2001). The spatial association between community air pollution and mortality: a new method of analyzing correlated geographic cohort data.. Environ Health Perspect.

[r7] Cao J, Yang C, Li J, Chen R, Chen B, Gu D (2011). Association between long-term exposure to outdoor air pollution and mortality in China: a cohort study.. J Hazard Mater.

[r8] Carey IM, Atkinson RW, Kent AJ, van Staa T, Cook DG, Anderson HR (2013). Mortality associations with long-term exposure to outdoor air pollution in a national English cohort.. Am J Respir Crit Care Med.

[r9] Census and Statistics Department, Government of the Hong Kong Special Administrative Region. (2002). Hong Kong 2001 Population Census: Basic Tables for Tertiary Planning Units.. http://www.statistics.gov.hk/pub/B11200242001XXXXB0400.pdf.

[r10] Census and Statistics Department, Government of the Hong Kong Special Administrative Region. (2011). Thematic Household Survey. Report No. 48.. http://smokefree.hk/UserFiles/resources/Statistics/Thematic_Household_Survey_No48.pdf.

[r11] Chau CK, Tu EY, Chan DW, Burnett J (2002). Estimating the total exposure to air pollutants for different population age groups in Hong Kong.. Environ Int.

[r12] Chen B, Kan H, Chen R, Jiang S, Hong C (2011). Air pollution and health studies in China—policy implications.. J Air Waste Manag Assoc.

[r13] Chen R, Kan H, Chen B, Huang W, Bai Z, Song G (2012). Association of particulate air pollution with daily mortality: the China Air Pollution and Health Effects Study.. Am J Epidemiol.

[r14] Crouse DL Peters PA van Donkelaar A Goldberg MS Villeneuve PJ Brion O 2012 Risk of nonaccidental and cardiovascular mortality in relation to long-term exposure to low concentrations of fine particulate matter: a Canadian national-level cohort study. Environ Health Perspect 120 708 714; 10.1289/ehp.1104049 22313724 PMC3346774

[r15] Dong GH, Zhang P, Sun B, Zhang L, Chen X, Ma N (2012). Long-term exposure to ambient air pollution and respiratory disease mortality in Shenyang, China: a 12-year population-based retrospective cohort study.. Respiration.

[r16] Hedley AJ, McGhee SM, Barron B, Chau P, Chau J, Thach TQ (2008). Air pollution: costs and paths to a solution in Hong Kong—understanding the connections among visibility, air pollution, and health costs in pursuit of accountability, environmental justice, and health protection.. J Toxicol Environ Health A.

[r17] Hedley AJ, Wong CM, Thach TQ, Ma S, Lam TH, Anderson HR (2002). Cardiorespiratory and all-cause mortality after restrictions on sulphur content of fuel in Hong Kong: an intervention study.. Lancet.

[r18] Hoek G Krishnan RM Beelen R Peters A Ostro B Brunekreef B 2013 Long-term air pollution exposure and cardio-respiratory mortality: a review. Environ Health 12 43; 10.1186/1476-069X-12-43 23714370 PMC3679821

[r19] Institute for the Environment, Hong Kong University of Science and Technology. (2013). Satellite Informatics System for Surface Particulate Matter Distribution.. http://envf.ust.hk/itf-si/.

[r20] Katanoda K, Sobue T, Satoh H, Tajima K, Suzuki T, Nakatsuka H (2011). An association between long-term exposure to ambient air pollution and mortality from lung cancer and respiratory diseases in Japan.. J Epidemiol.

[r21] Krewski D, Jerrett M, Burnett RT, Ma R, Hughes E, Shi Y (2009). Extended follow-up and spatial analysis of the American Cancer Society study linking particulate air pollution and mortality.. Res Rep Health Eff Inst.

[r22] Künzli N, Medina S, Kaiser R, Quénel P, Horak F, Studnicka M (2001). Assessment of deaths attributable to air pollution: should we use risk estimates based on time series or on cohort studies?. Am J Epidemiol.

[r23] Lam TH, Li ZB, Ho SY, Chan WM, Ho KS, Li MP (2004). Smoking and depressive symptoms in Chinese elderly in Hong Kong.. Acta Psychiatr Scand.

[r24] Li CC, Lau AKH, Mao JT, Chu DA (2005). Retrieval, validation, and application of the 1-km aerosol optical depth from MODIS measurements over Hong Kong.. IEEE Trans Geosci Remote Sens.

[r25] Ma R, Krewski D, Burnett RT (2003). Random effects Cox models: a Poisson modelling approach.. Biometrika.

[r26] Mahiyuddin WRW, Sahani M, Aripin R, Latif MT, Thach TQ, Wong CM (2013). Short-term effects of daily air pollution on mortality.. Atmos Environ.

[r27] NASA (National Aeronautics and Space Administration). (2013). NASA’s Earth Observing System Homepage.. http://eospso.nasa.gov/.

[r28] Neupane B, Jerrett M, Burnett RT, Marrie T, Arain A, Loeb M (2010). Long-term exposure to ambient air pollution and risk of hospitalization with community-acquired pneumonia in older adults.. Am J Respir Crit Care Med.

[r29] Nishiwaki Y, Michikawa T, Takebayashi T, Nitta H, Iso H, Inoue M (2013). Long-term exposure to particulate matter in relation to mortality and incidence of cardiovascular disease: the JPHC Study.. J Atheroscler Thromb.

[r30] Paciorek CJ, Liu Y, HEI Health Review Committee. (2012). Assessment and statistical modeling of the relationship between remotely sensed aerosol optical depth and PM_2.5_ in the eastern United States.. Res Rep Health Eff Inst.

[r31] Pope CA, Burnett RT, Thun MJ, Calle EE, Krewski D, Ito K (2002). Lung cancer, cardiopulmonary mortality, and long-term exposure to fine particulate air pollution.. JAMA.

[r32] R Core Team. (2013). R: A Language and Environment for Statistical Computing. Vienna, Austria:R Foundation for Statistcal Computing.. http://www.R-project.org.

[r33] Rajarathnam U Sehgal M Nairy S Patnayak RC Chhabra SK, Kilnani, et al. 2011 Part 2. Time-series study on air pollution and mortality in Delhi. Res Rep Health Eff Inst 157 47 74 21648204

[r34] Samoli E Peng R Ramsay T Pipikou M Touloumi G Dominici F 2008 Acute effects of ambient particulate matter on mortality in Europe and North America: results from the APHENA study. Environ Health Perspect 116 1480 1486; 10.1289/ehp.11345 19057700 PMC2592267

[r35] Ueda K, Nagasawa SY, Nitta H, Miura K, Ueshima H, NIPPON DATA80 Research Group. (2012). Exposure to particulate matter and long-term risk of cardiovascular mortality in Japan: NIPPON DATA80.. J Atheroscler Thromb.

[r36] WHO (World Health Organization). (2005). Health Effects of Transport-Related Air Pollution (Krzyzanowski M, Kuna-Dibbert B, Schneider J, eds). Copenhagen:WHO Regional Office for Europe.. http://www.euro.who.int/__data/assets/pdf_file/0006/74715/E86650.pdf.

[r37] WHO. (2010). International Statistical Classification of Diseases and Related Health Problems, 10th Revision.. http://apps.who.int/classifications/icd10/browse/2010/en.

[r38] Wong CM Vichit-Vadakan N Kan H Qian Z 2008 Public Health and Air Pollution in Asia (PAPA): a multicity study for short-term effects of air pollution on mortality. Environ Health Perspect 116 1195 1202; 10.1289/ehp.11257 18795163 PMC2535622

[r39] Zhang P Dong G Sun B Zhang L Chen X Ma N 2011 Long-term exposure to ambient air pollution and mortality due to cardiovascular disease and cerebrovascular disease in Shenyang, China. PLoS One 6 e20827; 10.1371/journal.pone.0020827 21695220 PMC3112212

[r40] Zhou M, Liu Y, Wang L, Kuang X, Xu X, Kan H (2014). Particulate air pollution and mortality in a cohort of Chinese men.. Environ Pollut.

