# Supplementary material for: Satellite-Based Estimates of Long-Term Exposure to Fine Particles and Association with Mortality in Elderly Hong Kong Residents
Source: Environ Health Perspect. 2015 Apr 24;123(11):1167–72. doi: 10.1289/ehp.1408264 (PMC4629733; doi:10.1289/ehp.1408264)
Supplement: (1.2 MB) PDF [file ehp.1408264.s001.acco.pdf]

Environ Health Perspect
doi: 10.1289/ehp.1408264

Note to Readers: *EHP* strives to ensure that all journal content is accessible to all readers. However, some figures and Supplemental Material published in *EHP* articles may not conform to 508 standards due to the complexity of the information being presented. If you need assistance accessing journal content, please contact ehp508@niehs.nih.gov. Our staff will work with you to assess and meet your accessibility needs within 3 working days.

Supplemental Material

Satellite-Based Estimates of Long-Term Exposure to Fine Particles and Association with Mortality in Elderly Hong Kong Residents

Chit Ming Wong, Hak Kan Lai, Hilda Tsang, Thuan Quoc Thach, G. Neil Thomas, Kin Bong Hubert Lam, King Pan Chan, Lin Yang, Alexis K.H. Lau, Jon G. Ayres, Siu Yin Lee, Wai Man Chan, Anthony J. Hedley, and Tai Hing Lam

Table of Contents

Figure S1. Scatterplot between annual $PM_{2.5}$ (4 stations) concentrations measured at general monitors and adjusted surface extinction coefficients at dry days (SECdry) (HKUST 2012) estimated at the same 1×1 km grid from NASA data year 2000–2011. *r* for the model = 0.60.Moderate Resolution Imaging Spectroradiometer (MODIS) data (http://modis.gsfc.nasa.gov/about/components.php) from two satellites, Terra (EOS AM) and Aqua (EOS PM) (http://modis.gsfc.nasa.gov/about/), were applied for correlation with aerosols and atmospheric optical conditions (http://disc.sci.gsfc.nasa.gov/giovanni/additional/users-manual/G3_manual_Chapter_8_MOVAS.shtml#What). Lidar measurement was used for estimating surface extinction coefficients (SEC) from aerosol optical depth (AOD), over non-reflective urban surface. SEC (unit: km-1) was controlled for humid and rainy day and stored as "SEC dry" data by the meteorological research team of the Institute of the Environment, The Hong Kong University of Science and Technology (http://envf.ust.hk/itf-si/).

Figure S2. Survival curves (Kaplan Meier) for each category of exposure to $PM_{2.5}$. The y-axis indicates the survival probability along the x-axis from the baseline to years of follow-up for

groups of individuals defined by quartiles of $PM_{2.5}$ exposure. The differences between groups were tested by log rank test (*p*-value <0.001).

Table S1. Hazard ratio (95% CI) of mortality from all natural causes adjusted for individual covariates only and together with ecological covariates measured at baseline 1998–2001.

Table S2. Hazard ratio of mortality per 10 μg/m^3 increase in $PM_{2.5}$ – stratified by education attainment

Table S3. Hazard ratio of mortality per 10 μg/m^3 increase in $PM_{2.5}$ – multi-level analyses.

Table S4. Hazard ratio of mortality per 10 μg/m^3 increase of $PM_{2.5}$ – adjustment for spatial autocorrelation.

Reference

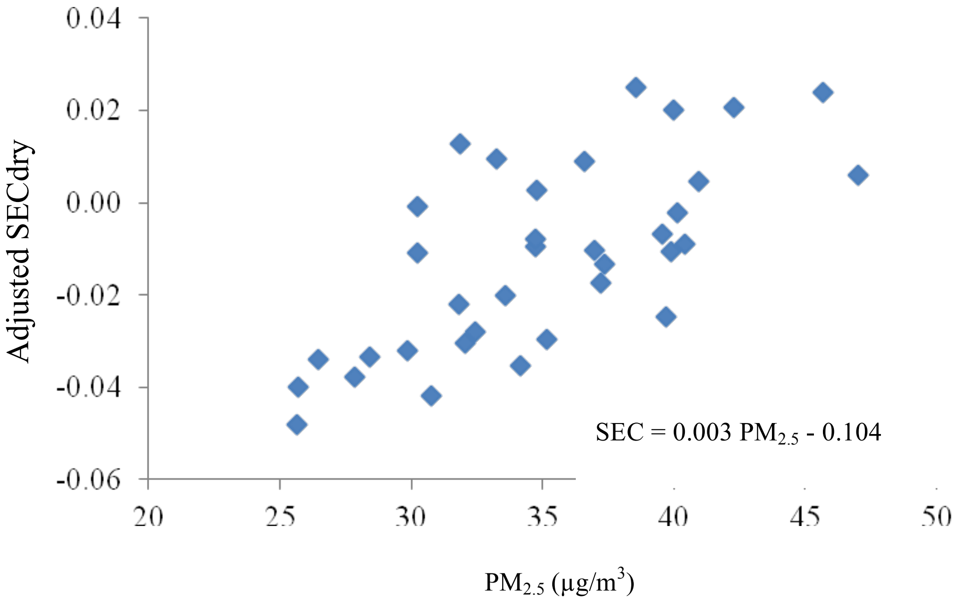

Figure S1. Scatterplot between annual $PM_{2.5}$ (4 stations) concentrations measured at general monitors and adjusted surface extinction coefficients at dry days (SECdry) (HKUST 2012) estimated at the same 1×1 km grid from NASA data year 2000–2011. *r* for the model = 0.60.Moderate Resolution Imaging Spectroradiometer (MODIS) data (http://modis.gsfc.nasa.gov/about/components.php) from two satellites, Terra (EOS AM) and Aqua (EOS PM) (http://modis.gsfc.nasa.gov/about/), were applied for correlation with aerosols and atmospheric optical conditions (http://disc.sci.gsfc.nasa.gov/giovanni/additional/users-manual/G3_manual_Chapter_8_MOVAS.shtml#What). Lidar measurement was used for estimating surface extinction coefficients (SEC) from aerosol optical depth (AOD), over non-reflective urban surface. SEC (unit: km-1) was controlled for humid and rainy day and stored as "SEC dry" data by the meteorological research team of the Institute of the Environment, The Hong Kong University of Science and Technology (http://envf.ust.hk/itf-si/).

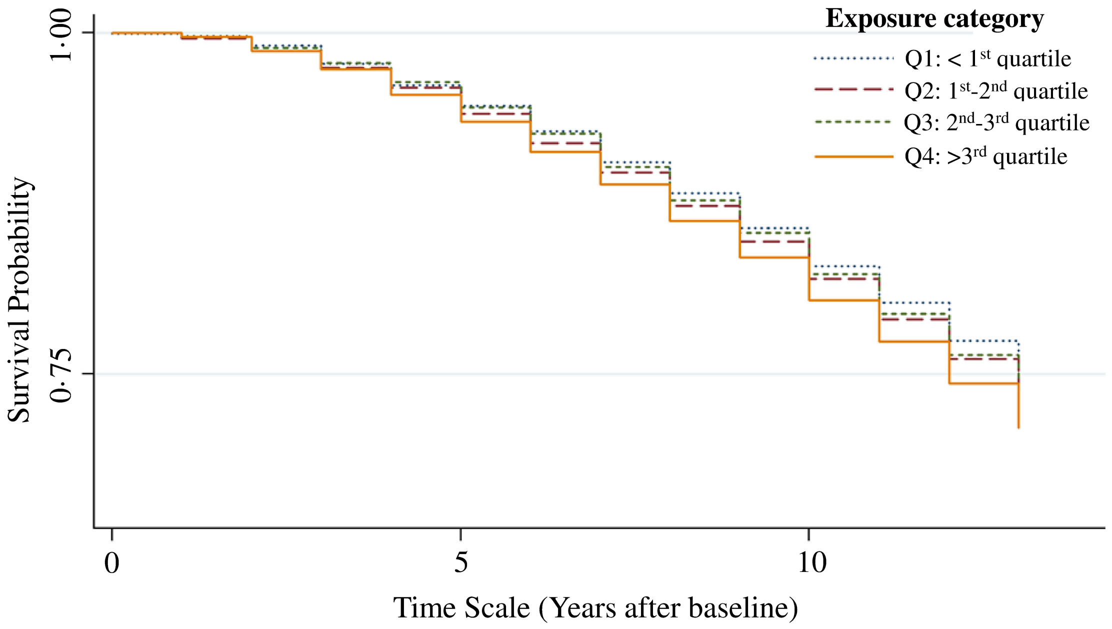

Figure S2. Survival curves (Kaplan Meier) for each category of exposure to $PM_{2.5}$. The y-axis indicates the survival probability along the x-axis from the baseline to years of follow-up for groups of individuals defined by quartiles of $PM_{2.5}$ exposure. The differences between groups were tested by log rank test (*p*-value <0.001).

Table S1. Hazard ratio (95% CI) of mortality from all natural causes adjusted for individual covariates only and together with ecological covariates measured at baseline 1998–2001.

**Variables**	**Individual covariates**	**Individual and ecological covariates**
Individual level:		
$PM_{2.5}$ concentration (per 10 μg/m^3)	1.13 (1.06, 1.21)***	1.14 (1.07, 1.22)***
Age (per year)	1.11 (1.11, 1.12)***	1.11 (1.11, 1.12)***
Gender: Male	1	1
Female	0.65 (0.63, 0.68)***	0.65 (0.63, 0.68)***
BMI quartiles: 2nd – 3rd (21.6–26.3)	1	1
<2nd (<21.6)	1.25 (1.21, 1.30)***	1.25 (1.21, 1.30)***
>3rd (>26.3)	1.03 (0.99, 1.07)	1.02 (0.98, 1.06)
Smoking: Never	1	1
Quitted	1.39 (1.33, 1.45)***	1.38 (1.32, 1.43)***
Current	1.73 (1.64, 1.81)***	1.70 (1.62, 1.79)***
Exercise (days per week)	0.97 (0.96, 0.98)***	0.97 (0.95, 0.98)***
Education: Secondary or above	1	1
Primary	1.11 (1.06, 1.16)***	1.09 (1.04, 1.14)***
Below primary	1.23 (1.17, 1.29)***	1.19 (1.13, 1.25)***
Expense per month (US$) <128	1.08 (1.02, 1.14)**	1.08 (1.02, 1.14)**
128–384	1.03 (0.99, 1.08)	1.03 (0.98, 1.07)
≥385	1	1
TPU level:		
Age≥65 (per %)	-	0.99 (0.98, 0.99)***
Education at tertiary level (per %)	-	0.99 (0.99, 1.00)***
Income/month ≥US$1,923 (per %)	-	1.00 (1.00, 1.00)[a]
District level:		
Smoking rate [as % of smokers] (per %)	-	1.06 (0.99, 1.14)

*p<0.05,**p<0.01,***p<0.001.

Crude HR per 10 μg/m^3 $PM_{2.5}$ concentration: 1.23 (1.16, 1.31) p<0.0001.

[a]The same HR (95%CI) but with three decimal points: 0.999 (0.996, 1.001).

Table S2. Hazard ratio of mortality per 10 μg/m^3 increase in $PM_{2.5}$ – stratified by education attainment

Education attainment	N	All natural causes	Interaction p-value	Cardio-respiratory	Interaction p-value
Secondary or above	10411	1.09(0.92,1.30)		1.09(0.84,1.41)	
Primary	22370	1.14(1.02,1.27)*	1.00	1.09(0.93,1.28)	0.75
Below primary	27446	1.17(1.06,1.29)**	0.46	1.23(1.07,1.42)**	0.36

*p<0.05, **p<0.01.

Table S3. Hazard ratio of mortality per 10 μg/m^3 increase in $PM_{2.5}$ – multi-level analyses.

Causes of death	Individual level	Multi-level analysis District + TPU	Multi-level analysis District + TPU + subject ID
All natural causes	1.14(1.07,1.22)***	1.14(1.07,1.22)***	1.14(1.07,1.22)***
Cardiovascular	1.21(1.07,1.37)**	1.21(1.07,1.38)**	1.21(1.07,1.38)**
- IHD	1.41(1.16,1.71)***	1.40(1.14,1.71)***	1.40(1.14,1.72)***
- Cerebrovascular	1.22(0.99,1.50)	1.23(1.00,1.52)	1.24(1.00,1.53)
Respiratory	1.04(0.89,1.21)	1.04(0.88,1.21)	1.05(0.89,1.23)
- Pneumonia	0.94(0.77,1.13)	0.93(0.76,1.13)	0.93(0.76,1.14)
- COPD	1.29(0.98,1.70)	1.29(0.96,1.71)	1.34(0.99,1.79)

R software was used for the analysis; *p<0.05, **p<0.01, ***p<0.001

Table S4. Hazard ratio of mortality per 10 μg/m^3 increase of $PM_{2.5}$ – adjustment for spatial autocorrelation.

Causes of death	No random effect	One-Level random effect in TPU, Distance-matrix (adjacency)	One-Level random effect in TPU, Distance-matrix (distance-decay in km)	Two-Level random effect in District and TPU, Distance-matrix (Distance decay within TPU)
All natural causes	1.14(1.07,1.22)***	1.14(1.07,1.22)***	1.14(1.07,1.22)***	1.14(1.07,1.22)***
Cardiovascular	1.22(1.08,1.39)**	1.21(1.07,1.37)**	1.21(1.07,1.37)**	1.20(1.06,1.37)**
- IHD	1.42(1.16,1.73)***	1.40(1.15,1.71)***	1.41(1.16,1.71)***	1.43(1.17,1.75)***
- Cerebrovascular	1.24(1.00,1.53)	1.22(0.99,1.50)	1.22(0.99,1.50)	1.24(1.00,1.53)
Respiratory	1.05(0.90,1.22)	1.04(0.89,1.21)	1.04(0.89,1.21)	1.05(0.90,1.23)
- Pneumonia	0.94(0.77,1.14)	0.93(0.77,1.13)	0.94(0.77,1.13)	0.94(0.77,1.15)
- COPD	1.30(0.98,1.74)	1.28(0.96,1.69)	1.28(0.96,1.68)	1.31(0.98,1.75)

R software was used for the analysis, the models were not adjusted for district level smoking rate.

*p<0.05, **p<0.01, ***p<0.001

Reference

Hong Kong University of Science and Technology (HKUST) 2012. Satellite Informatics System for Surface Particulate Matter Distribution. Institute of the Environment. Avaliable: http://envf.ust.hk/itf-si [accessed 27 March 2015].
